# Glycoprotein Structural Genomics: Solving the Glycosylation Problem

**DOI:** 10.1016/j.str.2007.01.011

**Published:** 2007-03

**Authors:** Veronica T. Chang, Max Crispin, A. Radu Aricescu, David J. Harvey, Joanne E. Nettleship, Janet A. Fennelly, Chao Yu, Kent S. Boles, Edward J. Evans, David I. Stuart, Raymond A. Dwek, E. Yvonne Jones, Raymond J. Owens, Simon J. Davis

**Affiliations:** 1 Nuffield Department of Clinical Medicine and MRC Human Immunology Unit, Weatherall Institute of Molecular Medicine, University of Oxford, Oxford OX3 9DS, United Kingdom; 2 Division of Structural Biology and Oxford Protein Production Facility, Wellcome Trust Centre for Human Genetics, University of Oxford, Oxford OX3 7BN, United Kingdom; 3 Oxford Glycobiology Institute, Department of Biochemistry, University of Oxford, South Parks Road, Oxford OX1 3QU, United Kingdom

## Abstract

Glycoproteins present special problems for structural genomic analysis because they often require glycosylation in order to fold correctly, whereas their chemical and conformational heterogeneity generally inhibits crystallization. We show that the “glycosylation problem” can be solved by expressing glycoproteins transiently in mammalian cells in the presence of the *N*-glycosylation processing inhibitors, kifunensine or swainsonine. This allows the correct folding of the glycoproteins, but leaves them sensitive to enzymes, such as endoglycosidase H, that reduce the *N*-glycans to single residues, enhancing crystallization. Since the scalability of transient mammalian expression is now comparable to that of bacterial systems, this approach should relieve one of the major bottlenecks in structural genomic analysis.

## Introduction

As early as 2003, structural genomics (SG) consortia were contributing almost a quarter of all new structures submitted to the Protein Data Bank (PDB; Terwilliger 2004). In addition to the rapid analysis of proteins that can be expressed and crystallized readily, the opportunity afforded by robotics and miniaturization to tackle difficult cases, using a variety of approaches in parallel, is likely to be a major benefit of the SG approach. A high level of attrition at each stage of all SG pipelines, however, is constraining their productivity to such an extent that, according to a recent analysis of the NIH Protein Structure Initiative (W. Minor; personal communication), only 2.5% of cloned genes are currently yielding structures. The two major bottlenecks are the production of soluble protein and of diffraction-quality crystals (Delucas et al., 2005). Glycoproteins present special problems because they often require glycosylation in order to fold correctly, precluding expression in bacteria (the most widely used expression host in current SG pipelines). Moreover, following expression in mammalian and insect cells, the chemical and conformational heterogeneity of their carbohydrate moieties generally inhibits crystallization. These factors probably explain why attention to, and success with, glycoprotein targets mean that only ∼10% of PDB entries, and 1% of the structures emerging from the NIH Protein Structure Initiative, are annotated as glycoproteins.

The dominant glycan modifications of proteins are initiated by the addition of GalNAc monosaccharides to the hydroxyl groups of serine or threonine residues, known as *O*-glycosylation, or by the transfer en bloc of a dolichol-linked oligosaccharide precursor to asparagines comprising Asn-X-Ser/Thr motifs, a process referred to as *N*-glycosylation (Gagneux and Varki, 1999). In each case, the glycans are extended and diversified by the successive activities of glycosidases and/or glycosyltransferases. In the context of protein crystallization, *O*-glycosylation constitutes less of a problem than *N*-glycosylation, since, in the great majority of cases, *O*-glycans are confined to extended, unfolded, serine-, threonine-, and proline-rich regions of polypeptide. These so-called STP domains are readily identified using *O*-glycosylation site-predicting algorithms, such as NetOGlyc (Julenius et al., 2005), or disorder-predicting algorithms, such as RONN (Yang et al., 2005), and can either be deleted from expression constructs or proteolytically cleaved at engineered sites following purification of the protein. Alternatively, these regions can often be deglycosylated using a combination of *O*-glycanase and neuraminidase (Leahy et al., 1992).

Our solution to the problem of *N*-glycosylation in a conventional crystallographic setting has relied on the use of stable mammalian expression systems, wherein folding and initial glycosylation proceed normally, but processing of the *N*-glycans is restricted in a way that allows their subsequent enzymatic removal with endoglycosidase (endo) H (Davis et al., 1993, 1995; Butters et al., 1999). Endo H cleaves between the GlcNAc residues in the di-*N*-acetylchitobiose core of oligomannose and hybrid-type *N*-glycans, leaving single GlcNAc residues at each glycosylation site. The Asn-linked GlcNAc is known to dominate glycan-protein interactions and to shield hydrophobic regions of the protein surface (Petrescu et al., 2004). We have found, with few exceptions, that the polydispersity in solution of endo H-treated proteins is indistinguishable from that of wild-type protein, whereas glycoproteins completely deglycosylated with, for example, PNGase F, tend to aggregate (Davis et al., 1995; J.A.F. and S.J.D., unpublished data).

We first produced endo H-sensitive proteins in cell lines generated from lectin-resistant Chinese hamster ovary (CHO) cells, such as *Lec3.2.8.1* cells, that are largely incapable of processing *N*-glycans beyond Man_5_GlcNAc_2_ intermediates (Davis et al., 1993). The analogous strategy of expressing glycoproteins in insect cells, which add paucimannose oligosaccharides sensitive to endo H and endo D, has since also been successful (Kwong et al., 1999). In an alternative strategy, we used the α-glucosidase inhibitor, *N*-butyldeoxynojirimycin (NB-DNJ), to block the very earliest stages of processing in cell lines generated from wild-type CHO cells, which also yielded glycoproteins with endo H-sensitive oligomannose *N*-glycans (Davis et al., 1995). The best results were obtained when the two approaches were combined (i.e., proteins were expressed in *Lec3.2.8.1* cells in the presence of NB-DNJ) (Butters et al., 1999). CHO cells are uniquely well suited to the use of NB-DNJ because they are largely deficient in a “shunt pathway,” wherein an endomannosidase circumvents α-glucosidase blockade, allowing downstream glycan processing (Moore and Spiro 1990, 1992; Hiraizumi et al., 1993). NB-DNJ is therefore unsuited to use in most, if not all, other mammalian expression systems. In the case of human embryonic kidney (HEK) 293T cells, for example, less than 5% of soluble (s) B7-1 expressed transiently in the presence of NB-DNJ proved to be endo H sensitive (K.S.B. and S.J.D., unpublished data).

Following the initial example of rat sCD2 (PDB accession no. 1hng), these approaches, in our hands and others, yielded structures of sCD58/CD2 (1ccz) and sCD48/CD2 chimeras (2dru), sB7-1 (1dr9), a soluble T-cell receptor (TCR) in complex with an anti-TCR Fab (1nfd), angiotensin-1-converting enzyme (1o8a), and murine sCD8αα (1bqh) and sCD8αβ (2atp). Non-endo H digested, *Lec3.2.8.1*-derived sICAM-1 (1ic1) and sICAM-2 (1zxq), sCEACAM1a (1l6z), soluble semaphorin 4D (1olz), and a sTCR/sMHC class-II complex (1d9k) also produced crystals, presumably due to the relative uniformity of *N*-glycosylation in *Lec3.2.8.1* cells.

Stable, mammalian cell-based protein expression cannot readily be implemented in a high-throughput setting because individual clones exhibit considerable variation in expression, necessitating clone selection. Because the yields, efficiency, and scalability of mammalian transient expression are each approaching those of high-throughput bacterial systems due to the advent of new episomal expression vectors, transfection protocols, and tissue culture methods (Durocher et al., 2002, Geisse and Henke 2005, Davies et al., 2005, Aricescu et al., 2006a, 2006c; Berntzen et al., 2005), we sought analogous methods for the production of endo H-sensitive glycoproteins in transiently transfected cells. In particular, we wanted to be able to produce endo H-sensitive proteins in HEK293 cells, which currently provide the benchmark for high-level, transient mammalian protein expression (Durocher et al., 2002; Berntzen et al., 2005).

We show here that glycoproteins transiently expressed in HEK293T cells in the presence of the *N*-glycosylation processing inhibitors, kifunensine or swainsonine, are highly sensitive to endo H. We also show that these inhibitors do not generally compromise overall expression yields. Since they target highly conserved processing enzymes that catalyze transformations essential for complex *N*-glycan formation downstream of the endomannosidase-dependent shunt pathway, it can be expected that these inhibitors will be effective in most mammalian cell-based expression systems.

## Results and Discussion

**Figure 1:**
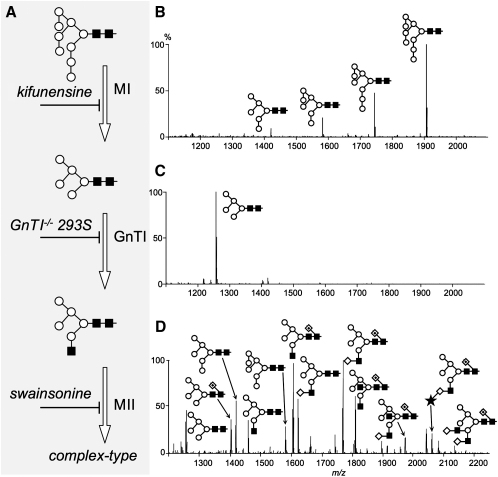
Manipulation of the Mammalian *N*-Linked Glycosylation Pathway in HEK293 Cells (A) A section of the glycosylation pathway is illustrated. (B–D) MALDI-TOF analysis of the glycans of the target glycoprotein (s19A) expressed in (B) HEK293T cells in the presence of the α-mannosidase I (MI) inhibitor, kifunensine, at 5 μM, resulting in the addition of oligomannose-type *N*-glycans, Man_5–9_GlcNAc_2_; (C) ricin-resistant HEK293S cells devoid of GnTI activity, resulting in predominantly Man_5_GIcNAc_2_*N*-glycans; and (D) HEK293T cells in the presence of the α-mannosidase II (MII) inhibitor, swainsonine, at 20 μM, resulting in the addition of hybrid-type glycans. For MALDI-TOF MS, glycans were released directly from SDS-PAGE gel bands by overnight PNGase F digestion. Monosaccharide constituents are represented as follows: open diamonds, Gal; closed diamonds, GaINAc; open squares, Glc; closed squares, GlcNAc; open circles, Man; stars, sialic acid; dotted diamonds, Fuc.

We expressed soluble forms of 19A, which is a stem cell marker protein (two IgSF domains, five glycosylation sites [Murphy et al., 2002]), as an initial test case, and the leukocyte cell surface antigen, CD48 (two IgSF domains, four glycosylation sites [Yokoyama et al., 1991]), and the receptor tyrosine phosphatase, RPTPμ (one MAM-, one IgSF-, and four FN3-domains, 12 glycosylation sites [Gebbink et al., 1993]), for replication purposes. Each protein was expressed transiently with either pEE14 (Bebbington and Hentschell 1987) or the commercial vector pEF-DEST51, which each contain the SV40 *ori* and the human cytomegalovirus and human elongation factor 1α promoter, respectively, or pHL, which contains the chicken β-actin promoter (Aricescu et al., 2006c). Endo H sensitivity was compared at two pH values, since the stabilities of some glycoproteins are pH sensitive (data not shown). An overview of mammalian *N*-glycan processing and the effects of our strategies revealed by MALDI-TOF MS are shown in Figure 1.

**Figure 2:**
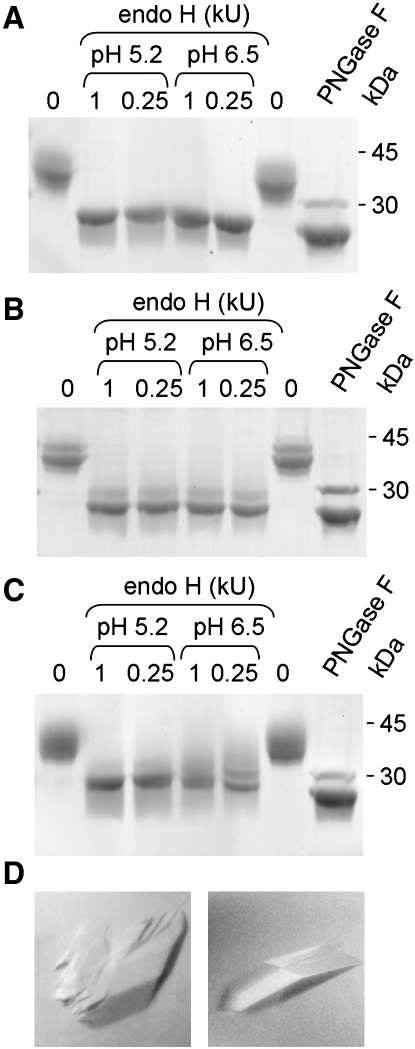
Endo H Digestion of s19A Produced in HEK293 Cells under Various Conditions (A–C) SDS-PAGE gels, run under reducing conditions, of endo H- or PNGase F-treated s19A expressed in (A) GnTI-deficient HEK293S cells, (B) HEK293T cells cultured with 5 μM kifunensine, and (C) HEK293T cells cultured with 20 μM swainsonine. In each case, 5 μg of purified s19A was treated at 37°C with 1 kU or 0.25 kU of endo H at the indicated pH, or at 37°C with 0.5 kU of PNGase F at pH 7.4, for 6 hr. For the endo H digests, identity of the products indicates that the digestions have gone to completion. (D) Crystals of endo H-treated sRPTPμ expressed in GnTI-deficient HEK293S cells (left panel) and endo H-treated s19A expressed in HEK293T cells in the presence of swainsonine (right panel). See Supplemental Data for the endo H-sensitivity and SDS-PAGE analysis of sRPTPμ. Data have been collected in space groups C2 and P2_1_2_1_2_1_ for RPTPμ and s19A, respectively (see Experimental Procedures).

We first investigated the use of a ricin-selected, *N*-acetylglucosaminyltransferase I (GnTI)-deficient HEK293S-derived cell line used previously for the stable expression of rhodopsin with Man_5_GlcNAc_2_ adducts (Reeves et al., 2002). GnTI catalyzes the formation of hybrid-type glycans via transfer of β1-2-linked GlcNAc to the 3-antenna of the oligomannose substrate, Man_5_GlcNAc_2_ (Figure 1A). This is required for formation of complex-type glycans, as it allows cleavage of the 6-antennae mannoses by Golgi α-mannosidase II. Transiently transfected, GnTI-deficient HEK293S cells secreted s19A bearing only Man_5_GlcNAc_2_-type *N*-glycans (Figure 1C). This protein proved to be very sensitive to endo H at pH 5.2 and 6.5 (Figure 2A), as did sRPTPμ (Crispin et al., 2006) and sCD48 (see Figures S1 and S2, gel A, in the Supplemental Data available with this article online). The extreme endo H sensitivity of GnTI-deficient, HEK293S-derived material contrasts with that of glycoproteins from GnTI-deficient CHO cells (i.e., *Lec1* cells: <50% sensitivity; data not shown), or from CHO cells lacking three additional processing enzymes (i.e., *Lec3.2.8.1* cells: 50%–70% sensitivity [Butters et al., 1999]). This suggests that HEK293S cells lack an α-mannosidase activity that is present in CHO cells (Crispin et al., 2006). Furthermore, in contrast to proteins expressed in CHO *Lec3.2.8.1* cells, GnTI-deficient 293S-derived glycoproteins seem to contain only traces of core fucose (Crispin et al., 2006), further enhancing endo H cleavage. Crystals diffracting beyond 3 Å grew from endo H-treated sRPTPμ expressed in GnTI-deficient HEK293S cells (Figure 2D, left panel), whereas crystals of the fully glycosylated protein only diffracted to a Bragg spacing of >8 Å.

These observations suggest that GnTI-deficient HEK293S cells could, in principle, be used as a platform for the high-throughput production of deglycosylatable glycoproteins. We found, however, that expression in these cells is only 10%–50% as high as that obtainable in HEK293T cells, regardless of which expression vector is used or whether the SV40 large T antigen, which is stably expressed by 293T cells and favors expression from SV40 *ori*-containing plasmids, is present (data not shown). We therefore investigated the possibility that *N*-glycan processing inhibitors other than NB-DNJ might be effective in 293T cells, taking advantage of the enhanced expression capability of these cells.

The alkaloids, kifunensine and swainsonine, which are potent inhibitors of α-mannosidases I and II, respectively (Figure 1A), were chosen for analysis because they act downstream of the endomannosidase-mediated shunt pathway likely to be responsible for the poor yields of endo H-sensitive protein from HEK293T cells treated with NB-DNJ (K.S.B. and S.J.D., unpublished data). Somewhat unexpectedly, 293T cells cultured with kifunensine and swainsonine produced larger quantities of s19A than 293T cells alone (by ∼30% and 10%, respectively; data not shown). In the case of kifunensine, it is likely that this is due to its inhibition of ER-associated degradation (Tokunaga et al., 2003). *N*-glycans released from s19A expressed in the presence of kifunensine and swainsonine were of the oligomannose- and hybrid-types, respectively (Figures 1B and 1D). The complexity of the swainsonine-induced hybrid-type structures (Figure 1D) reveals the presence of the GnTI-dependent activities of several glycosyltransferases, including GnTIII, which adds bisecting β1-4GlcNAc, and FUT8, which catalyzes the transfer of α1-6-linked fucose to the reducing terminal GlcNAc. Endo H treatment of s19A (Figures 2B and 2C), sRPTPμ (Figure S1), and sCD48 (Figure S2) secreted in the presence of both inhibitors yielded essentially glycan-free material at pH 5.2. The digestion of sRPTPμ and sCD48 at pH 6.5 was less effective for swainsonine- than for kifunensine-derived material (Figures S1 and S2, gels B and C), reflecting the lower endo H susceptibility of core α1-6 fucosylated hybrid-type glycans and the stringent inhibition of core fucosylation by kifunensine (Crispin et al., 2006). s19A, secreted in the presence of swainsonine, crystallized (Figure 2D, right panel), and these crystals diffracted to <3 Å; we have yet to obtain crystals of s19A from kifunensine-treated cultures. Total sCD48 recovery following endo H digestion was higher at pH 6.5 than at pH 5.2 (Figure S2), signaling the pH sensitivity of this protein (crystallization of sCD48 has not been attempted).

Our results show that kifunensine and swainsonine can be used to prepare deglycosylatable glycoproteins in 293T cells, without compromising protein yield. There is every reason to expect that these inhibitors will be effective in most mammalian expression systems, given (1) their activity downstream of the endomannosidase shunt pathway, and (2) the highly conserved structures of the active sites and inhibitor-binding properties of α-mannosidase I and II from organisms as diverse as mammals, insects, and yeast (Kawar et al., 2000; van den Elsen et al., 2001; Tempel et al., 2004). We can already confirm that kifunensine is effective in transiently transfected 293E cells (Durocher et al., 2002) and stably transfected CHO-K1 cells (Davis et al., 1990; data not shown). Kifunensine has the practical advantage over swainsonine of being active at 2- to 4-fold-lower concentrations, making it much more cost effective. Moreover, treatment with kifunensine leads to a much more homogeneous product, since it is better at suppressing the formation of core-fucosylated hybrid structures, which are somewhat endo H resistant. For most proteins, this is likely to be a significant advantage. However, in the case of s19A, we obtained crystals of endoH-treated protein from swainsonine-treated but not kifunensine-treated cultures, suggesting that the fucose residues remaining after removal of the bulk of each glycan can contribute to the crystallizability of the protein. Therefore, both inhibitors could be trialed for the crystallization of recalcitrant proteins.

It is worth noting that other approaches for expressing deglycosylatable glycoproteins, or glycoproteins with minimal or no glycosylation, also exist. The yeast, *Pichia pastoris*, can be used to express glycoproteins with endo H-sensitive *N*-glycans, although, in our experience, certain classes of mammalian proteins fail to fold efficiently in these cells (S.J.D., unpublished data; Aricescu et al., 2006a). Expression in insect cells (e.g., *S. frugiperda* [*Sf9*]) has the advantage that compact *N*-glycans dominated by the fucosylated Man_3_GlcNAc_2_Fuc paucimannose hexasaccharide are added (Figure S3A). This structure lacks the extreme structural and conformational heterogeneity characteristic of mammalian complex-type *N*-glycans, accounting for the many structures of fully glycosylated, insect cell-expressed proteins that have been published (e.g., Choe et al., 2005). Endo D and endo H have also been used in combination to reduce the Man_3_GlcNAc_2_Fuc hexasaccharide and residual oligomannose-type glycans, respectively, to single, potentially fucosylated, GlcNAc residues on HIV-1 gp120 (Kwong et al., 1999), an observation we now extend to sRPTPμ (Figure S3A). Significant problems with this approach, however, are the time scales and expense of insect cell culture, and, in our experience, protein yields that are at least several-fold lower than those obtainable via transient expression in HEK293T cells. sRPTPμ, for example, is expressed at 1 mg/l and 35 mg/l in *Sf9* and HEK293T cells, respectively (data not shown). Finally, mutation of glycosylation sites prior to expression has facilitated the crystallization of, among other proteins, the ADP-ribosyl cyclase CD157 (1isf), Zn-α_2_-glycoprotein (1t7v), butyrylcholinesterase (1xlw), angiotensin I-converting enzyme (2iul, 2iux), and procathepsin (1mir). In several cases, a subset of the glycosylation sites had to be left intact in order for these proteins to fold correctly. A complementary strategy for identifying nonessential glycosylation sites, by virtue of their being variably occupied in the native protein, has recently been described (Nettleship et al., 2007).

It has been argued that SG methodologies could be broadened to better accommodate targets of higher technical difficulty and greater scientific “impact” (Aricescu et al., 2006a; Chandonia and Brenner 2006). The methods we present ought to facilitate the analysis of glycoproteins, not only in general structural biology laboratories, but also by bringing this important class of molecule within reach of existing SG pipelines.

## Experimental Procedures

### Preparation of Constructs

A cDNA fragment encoding the sRPTPμ extracellular region (residues 1–724) was amplified by polymerase chain reaction (PCR) with the phFL vector as template (a gift from M. Gebbink, University of Utrecht, The Netherlands [Gebbink et al., 1993]) and subcloned into the pHL expression vector (Aricescu et al., 2006b) and pFastBac1 (Invitrogen Ltd, Paisley, UK) in frame with a C-terminal LysHis_6_ tag. PCR was also used to amplify cDNA fragments encoding the human s19A and sCD48 extracellular regions (residues 1–222 and 1–223, respectively) and C-terminal LysHis_6_ tags from plasmid templates (M.T. Vuong, personal communication). The genes were inserted into the expression vectors pEF-DEST51 (Invitrogen Ltd, Paisley, UK) by Gateway cloning, pHL (Aricescu et al., 2006c), or pEE14 (Bebbington and Hentschell, 1987).

### Expression in HEK293T Cells

HEK293T (ATCC no. CRL-1573) and GnTI-deficient HEK293S cells (a gift from H.G. Khorana and P.J. Reeves, Massachusetts Institute of Technology [Reeves et al., 2002]), each cultured as adherent monolayers in Dulbecco's modified Eagle's medium (Sigma-Aldrich Company Ltd., Gillingham, UK) supplemented with 10% fetal calf serum (v/v; Sigma-Aldrich Company Ltd.), L-glutamine and nonessential amino acids (Invitrogen Ltd., Paisley, UK), were transiently transfected with the “25 kDa branched” form of polyethyleneimine (PEI; Sigma-Aldrich Company Ltd.) (Durocher et al., 2002; Kichler 2004; Demeneix and Behr 2005). Briefly, the PEI was diluted to 1 mg/ml from a stock solution of 100 mg/ml and pH-adjusted to pH 7 with HCl. The plastic-adherent cells were grown to ∼90% confluency in 3–4 Nunc TripleFlask tissue culture flasks (3 × 176 cm^2^/flask surface area; Cole-Parmer Instrument Company Ltd., Hanwell, UK) in 120 ml of medium per flask. Plasmid DNA (150 μg/flask) was added to 5 ml of serum-free medium and mixed with 300 μl/flask of PEI (1 mg/ml), followed by brief vortexing. The solution was incubated for 10 min at room temperature to allow DNA-PEI complex formation. During complex formation, medium from the plates to be transfected was replaced with 120 ml/flask of fresh medium containing swainsonine (Gross et al., 1983) or kifunensine (Toronto Research Chemicals, North York, ON, Canada) (Elbein et al., 1990) at final concentrations of 20 μM and 5 μM, respectively, and fetal calf serum at a concentration of 2% (v/v). Finally, the DNA-PEI complex was added to the flask, with brief rotation to allow mixing. Four to five days later, the supernatant was harvested for protein purification.

### Glycoprotein Purification

Tissue culture supernatant, typically up to 0.5 liter, containing the secreted protein, was harvested and debris removed by centrifugation at 5000 × g at 4°C for 20 min. An equal volume of PBS (pH 8.0), 0.05% NaN_3_, was added, along with 2–4 ml/l of a 50% slurry of Ni-NTA agarose beads (QIAGEN, West Sussex, UK). The mixture was gently stirred at 4°C overnight and the beads collected by centrifugation at 200 × g for 5 min in 50 ml tubes (Falcon, BD Biosciences, Oxford, UK) at 4°C. The beads were poured into an EconoColumn (Bio-Rad Laboratories Ltd., Hemel Hempstead, UK) and washed with 10 column volumes of PBS (pH 8.0) prior to pre-elution with 2 volumes of 10 mM imidazole (pH 8.0) and elution with 2 volumes of 250 mM imidazole (pH 8.0). Eluting fractions containing protein of the expected size, according to SDS-PAGE analysis, were pooled and concentrated to ∼300 μl for further purification by Sephacryl S-200 gel filtration in 10 mM HEPES (pH 7.4), 150 mM NaCl. Fractions containing the proteins at >95% purity, according to SDS-PAGE analysis, were pooled and stored at 4°C.

### Protein Deglycosylation

The purified proteins were deglycosylated with endo H_f_ (New England Biolabs, Hitchin, UK). Trial digests of 5 μg of protein at 37°C, with a series of 4-fold dilutions of endo H_f_ starting with 500 U of the enzyme in a final volume of 20 μl of 0.1 M NaOAc at pH 5.2, were used to estimate the amount of endo H_f_ required for full deglycosylation. Generally, the trial incubations were done for 1 hr and 3 hr in order to limit the amount of time that larger amounts of the protein would eventually have to be kept at low pH (i.e., pH 5.2). The extent of digestion was monitored by SDS-PAGE analysis; identity of the 1 hr and 3 hr samples was taken to indicate that digestion had gone to completion within 1 hr. For highly pH-sensitive protein (i.e., protein that visibly precipitates during the course of the digestion at pH 5.2 [e.g., sRPTPμ and sCD48]), longer digestions at the nonoptimal pH 7.4 were used, necessitating larger amounts of enzyme. For large-scale digestion of s19A expressed in the presence of swainsonine, 0.6 mg of protein at 0.17 mg/ml in 0.1 M NaOAc (pH 5.2) was digested with 35.7 kU of endo H_f_ for 1 hr at 37°C. For the digestion of sRPTPμ expressed in GnTI-deficient HEK293S cells, 2.3 mg of protein at 1.5 mg/ml in 10 mM HEPES (pH 7.4), 150 mM NaCl, was digested with 92 kU of endo H_f_ for 6 hr at 37°C. Following digestion at low pH (i.e., pH 5.2), the samples were neutralized with an equal volume of 1 M Tris (pH 8.0). Prior to further purification, the digested samples were centrifuged at 5000 × g for 5 min to remove any precipitates, and then concentrated to 0.4–0.5 ml. Deglycosylated protein was finally separated from endo H_f_-resistant protein by Sephadex S-200 gel filtration in 10 mM HEPES (pH 7.4), 150 mM NaCl. Overall yields of deglycosylated protein were in the region of 25%–50%.

### Crystallization

Crystals of sRPTPμ, expressed in GnTI-deficient 293S cells and deglycosylated with endo H_f_, were grown by sitting-drop vapor diffusion at 22°C. Droplets (100 nl) of protein at 3.7 mg/ml in 10 mM HEPES (pH 7.4), 150 mM NaCl, mixed with 100 nl of reservoir solution, were set in 96-well plates (Greiner Bio-One Ltd, Stonehouse, UK) by a Cartesian Technologies MIC4000 robot, as previously described (Walter et al., 2005). Crystals in space group C2 (a = 167.3 Å, b = 69.3 Å, c = 97.5 Å, β = 113.5°) appeared after 18 days against a reservoir containing polyethylene glycol SMEAR (22.5% w/v) (Newman et al., 2005), 100 mM bis-Tris propane (pH 6.5), and 200 mM potassium thiocyanate. Crystals were cryoprotected with perfluoropolyether oil (PFO-X125/03; Lancaster Synthesis, Inc., Windham, NH), and data were collected at beamline ID29 (ESRF, Grenoble, France). Diffraction data to 3 Å with 99.5% completeness and an R_merge_ of 17.4% were collected.

Crystals of s19A, expressed in HEK293T cells with 20 μM swainsonine and deglycosylated with endo H_f_, were grown by sitting-drop vapor diffusion at 22°C. Droplets (100 nl) of this protein at 6 mg/ml in 10 mM HEPES (pH 7.4), 150 mM NaCl, mixed with 100 nl of reservoir solution, were also set in 96-well plates by the Cartesian Technologies MIC4000 robot. Crystals in space group P2_1_2_1_2_1_ (a = 64.75 Å, b = 55.12 Å, c = 156.67 Å; α = β = γ = 90°) appeared against a reservoir containing 10 mM magnesium chloride, 30% v/v polyethylene glycol 400, 100 mM potassium chloride in 50 mM Tris-HCl (pH 8.5), with pH adjustment according to Walter et al. (2005). Diffraction data to 2.6 Å with 97.1% completeness and an R_merge_ of 12.0% were collected.

### Release of *N*-Linked Glycans by Protein *N*-Glycanase F for MALDI-TOF Analysis

Coomassie blue-stained bands containing approximately 10 μg of target glycoprotein were excised from reducing SDS-PAGE gels and washed with 20 mM NaHCO_3_ (pH 7.0). The washed gel bands were dried in a vacuum centrifuge before rehydration in 30 μl of 30 mM NaHCO_3_ (pH 7), containing 100 U/ml of protein *N*-glycanase F (PNGase F; Glyko Inc., Novato, CA, USA). After incubation for 12 hr at 37°C, the enzymatically released *N*-linked glycans were eluted with water. Salts were removed by incubation at room temperature (5 min) with 200 μl of an acid-activated AG-50W slurry (200–400 mesh; Bio-Rad), which was removed by filtration with a 0.45 μl pore-size filter (Millex-LH, hydrophobic polytetrafluoroethylene).

### MALDI-TOF Mass Spectrometry of *N*-Linked Glycans

Positive ion MALDI-TOF mass spectra were recorded with a Waters-Micromass (Manchester, UK) TofSpec 2E reflectron-TOF mass spectrometer operated under the following conditions: accelerating voltage, 20 kV; pulse voltage, 3.0 kV; time-lag focusing delay, 500 ns (setting 39); laser repetition rate, 10 Hz. Aqueous glycan samples (0.5 μl) were mixed on the MALDI target with the matrix (0.5 μl of a saturated solution of 2,5 dihydroxybenzoic acid in acetonitrile), allowed to dry under ambient conditions, and recrystallized from ethanol (0.2 μl). Structural assignments of all MS spectra were made by composition, based on the characteristic fragmentation of molecular ions by collision-induced decomposition (Harvey 2005).

## Supplemental Data

Document S1. Three Figures and Supplemental Experimental Procedures
