# Supplementary material for: Glycoprotein Structural Genomics: Solving the Glycosylation Problem
Source: Structure. 2007 Mar;15(3):267–73. doi: 10.1016/j.str.2007.01.011 (PMC1885966; doi:10.1016/j.str.2007.01.011)
Supplement: Document S1. Three Figures and Supplemental Experimental Procedures [file mmc1.pdf]

Structure *15*

Supplemental Data

Glycoprotein Structural Genomics: Solving the Glycosylation Problem

Veronica T. Chang, Max Crispin, A. Radu Aricescu, David J. Harvey, Joanne E. Nettleship, Janet A. Fennelly, Chao Yu, Kent S. Boles, Edward. J. Evans, David I. Stuart, Raymond A. Dwek, E. Yvonne Jones, Raymond J. Owens, and Simon J. Davis

Supplemental Experimental Procedures

Construction of Baculoviruses and Expression in *Sf9* Cells

Baculovirus expressing the RPTPμ extracellular region were constructed using the Bac-to-Bac® method according to the manufacturer's protocol. Primary virus stocks were amplified and used to infect 1 to 5L suspension cultures of *Sf9* cells in SF900II medium (InVitrogen Ltd, Paisley, UK) at a multiplicity of infection of approximately 5. Cultures (2 L) were grown in Erlenmeyer flasks at 37°C.

Release of *N*-Linked Glycans by β-Elimination

As insect cell-derived glycoproteins can potentially contain core α1−3 fucose, which prevents PNGase F cleavage, glycoproteins from the bacuolovirus expression system were released by β-elimination. Purified glycoproteins (50 μg) from the baculovirus expression system were dried and dissolved in 1 mL of saturated ammonium carbonate solution in 29% ammonium hydroxide. After incubation at 60°C for 40 hrs, the reaction mixture was evaporated to dryness. The ammonium hydroxide and ammonium carbonate were removed by dissolving the sample in 1 mL water, evaporating to dryness. The dried sample was dissolved in 100 μL of 0.5 M boric acid and incubated for 30 min at 37°C. The solution was evaporated to dryness and further boric acid was removed by two cycles of 1 mL methanol addition and evaporation. The sample was then dissolved in 100 μL of water. An aliquot of the released glycans was derivatised with 2-aminobenzamide (2AB) according to Bigge *et al.* (1995), to facilitate HPLC analysis (data not shown).

Supplemental Reference

Bigge, J.C., Patel, T.P., Bruce, J.A., Goulding, P.N., Charles, S.M., and Parekh, R.B. (1995). Nonselective and efficient fluorescent labeling of glycans using 2-amino benzamide and anthranilic acid. Anal. Biochem. *230*, 229-238.

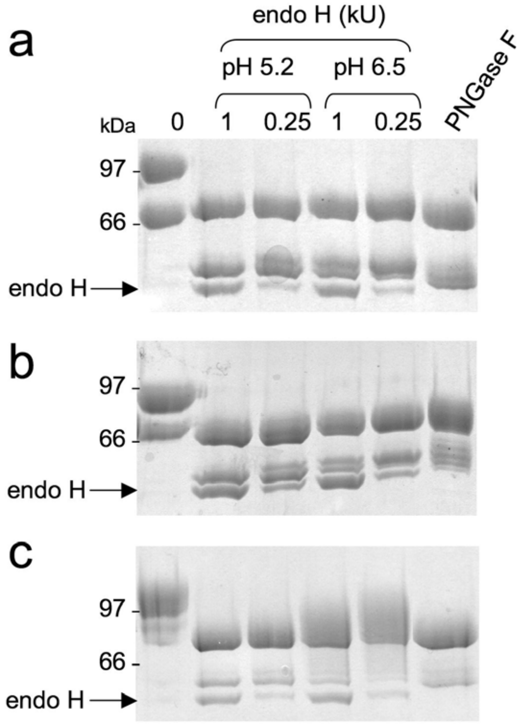

Figure S1. Endo H-Sensitivity of sRPTPμ Expressed in HEK 293 Cells

SDS-PAGE gels, run under reducing conditions, of endo H or PNGase F-treated sRPTPμ expressed in (**A**) GnTI-deficient HEK 293S cells, (**B**) HEK 293T cells cultured with 5 μM kifunensine and (**C**) HEK 293T cells in the presence of 20 μM swainsonine. In each case, 5 μg of purified sRPTPμ was treated at 37°C with 1 kU or 0.25 kU of endo H at the indicated pH, or at 37°C with 0.5 kU of PNGase F at pH 7.4, for 6 h. The multiple bands present in the sRPTPμ samples are common to the PTP family and arise from proteolytic 'nicking'. The larger, un-nicked fragment, when deglycosylated, is approximately the same size as the glycosylated nicked fragments.

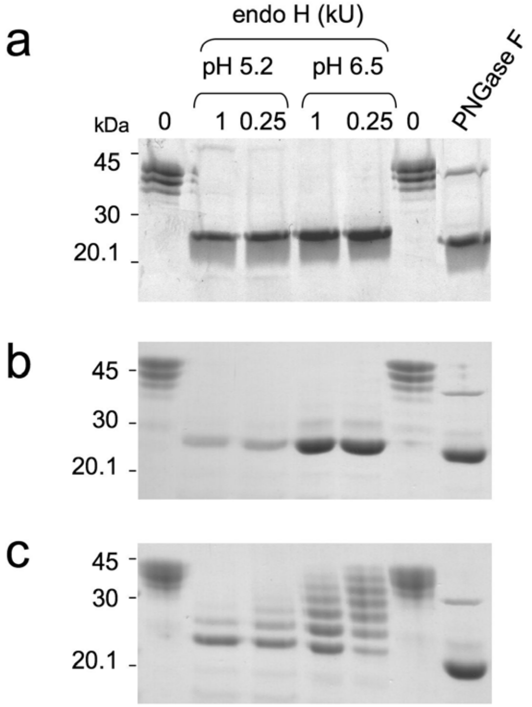

Figure S2. Endo H-Sensitivity of sCD48 Expressed in HEK 293 Cells

SDS-PAGE gels, run under reducing conditions, of endo H or PNGase F-treated CD48 expressed in (**A**) GnTI-deficient HEK 293S cells, (**B**) HEK 293T cells grown in 5 µM kifunensine and (**C**) HEK 293T cells in the presence of 20 µM swainsonine. In each case, 5 µg of purified sCD48 was treated at 37°C with 1 kU or 0.25 kU of endo H at the indicated pH, or at 37°C with 0.5 kU of PNGase F at pH 7.4, for 6 h.

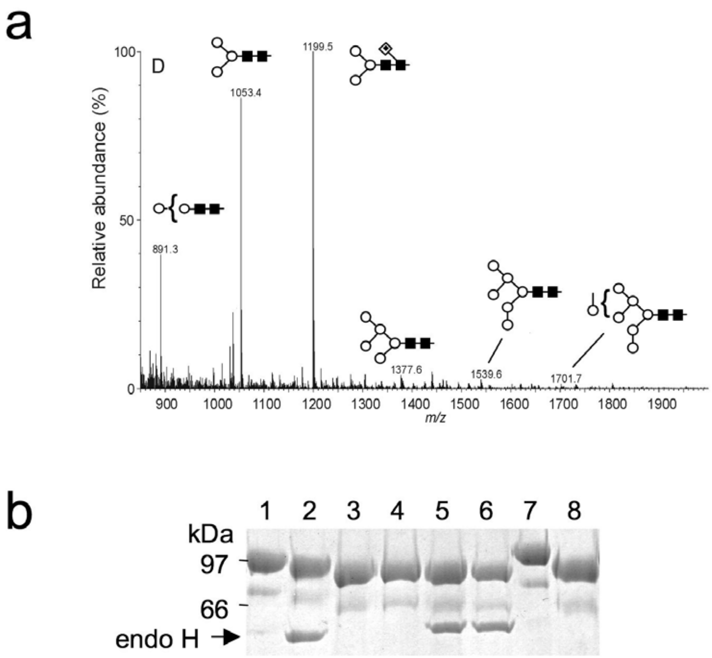

Figure S3. Deglycosylation of sRPTPμ Expressed in the Bacuolovirus System

(**A**) MALDI-TOF MS spectra of glycans released by β-elimination and derivatized with 2-aminobenzamide, and (**B**) SDS-PAGE gel showing the sensitivity of the protein to endoglycosidase digestion. Deglycosylation of 20 μg of undenatured sRPTPμ expressed in *Sf9* insect cells was carried out with the following enzymes for 6 h: *lane 1*, undigested; *lane 2*, 1 kU of endo H at pH 6.5; *lane 3*, 6 mU of endo D at pH 6.5; *lane 4*, 4 mU of Endo F_3 at pH 5.2; *lane 5*, endo H and endo D; *lane 6*, endo H and endo F_3; *lane 7*, undigested. For comparison, denatured sRPTPμ was also digested with 0.5 kU of PNGase F at pH 7.4 (*lane 8*).
